# Therapeutic perspective of thymoquinone: A mechanistic treatise

**DOI:** 10.1002/fsn3.2070

**Published:** 2021-01-28

**Authors:** Masood Sadiq Butt, Muhammad Imran, Ali Imran, Muhammad Sajid Arshad, Farhan Saeed, Tanweer Aslam Gondal, Mohammad Ali Shariati, Syed Amir Gilani, Tabussam Tufail, Ishtiaque Ahmad, Nadir Ali Rind, Mohamad Fawzi Mahomoodally, Saiful Islam, Zaffar Mehmood

**Affiliations:** ^1^ Faculty of Food, Nutrition & Home Sciences National Institute of Food Science and Technology UAF Faisalabad Pakistan; ^2^ Faculty of Allied Health Sciences University Institute of Diet and Nutritional Sciences The University of Lahore Lahore Pakistan; ^3^ Department of Food Science Institute of Home and Food Sciences Government College University Faisalabad Pakistan; ^4^ School of Exercise and Nutrition Faculty of Health Deakin University Burwood Vic. Australia; ^5^ Food Engineering Department Shakarim State University of Semey Semey Kazakhstan; ^6^ Department of Dairy Technology University of Veterinary and Animal Sciences Lahore Pakistan; ^7^ Department of molecular Biology and Genetics Shaheed Benazir Bhutto University Shaheed Benazirabad Pakistan; ^8^ Department of Health Sciences Faculty of Medicine and Health Sciences University of Mauritius Réduit Mauritius; ^9^ Institute of Nutrition and Food Science University of Dhaka Dhaka Bangladesh; ^10^ School of life Sciences Forman Christian College (A Chartered University) Lahore Pakistan

**Keywords:** anticancer, antioxidant potential, cardioprotective disorder, phytochemical, thymoquinone

## Abstract

The higher utilization of fruits and vegetables is well known to cure human maladies due to the presence of bioactive components. Among these compounds, thymoquinone, a monoterpene and significant constituent in the essential oil of *Nigella sativa L*., has attained attention by the researchers due to their pharmacologies perspectives such as prevention from cancer, antidiabetic and antiobesity, prevention from oxidative stress and cardioprotective disorder. Thymoquinone has been found to work as anticancer agent against different human and animal cancer stages including propagation, migration, and invasion. Thymoquinone as phytochemical also downregulated the Rac1 expression, mediated the miR‐34a upregulation, and increased the levels of miR‐34a through p53, as well as also regulated the pro‐ and antiapoptotic genes and decreased the phosphorylation of NF‐κB and IKKα/β. In addition, thymoquinone also lowered the metastasis and ERK1/2 and PI3K activities. The present review article has been piled by adapting narrative review method and highlights the diverse aspects of thymoquinone such as hepatoprotective, anti‐inflammatory, and antiaging through various pathways, and further utilization of this compound in diet has been proven effective against different types of cancers.

## Graphical abstract

Thymoquinone is strong anticancer agent through multiple mechanistic routes.

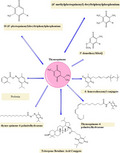

## 1 INTRODUCTION

The higher utilization of fruits, vegetables, spices, and herbs has been used as practical strategy to prevent from the different types of human cancers and other maladies such as diabetes, cardiovascular disease, obesity due to the presence of bioactive compounds. Among these phytochemicals, thymoquinone from *Nigella sativa* has been found to be very effective against various human maladies such as cancer insurgence, diabetes prevalence, obesity, inflammation, cardiovascular disease, and oxidative stress, as well as also several infectious microbial diseases. It also prevents the cardiotoxicity induced by doxorubicin through suppression of the carcinogenesis activity, membrane lipid peroxidation, destruction of the Fe‐NTA‐induced oxidative stress, and inhibition of the eicosanoid production (Farah & Begum, 2003; Yimer et al., 2019). *Nigella sativa* has attracted healers in ancient civilizations and researchers in recent times. Traditionally, it has been used in different forms to treat many diseases including asthma, hypertension, diabetes, inflammation, cough, bronchitis, headache, eczema, fever, dizziness, and influenza. Experimentally, it has been demonstrated that *N. sativa* extracts and the main constituent of their volatile oil, thymoquinone, possess antioxidant, anti‐inflammatory, and hepatoprotective properties (Khader & Eckl, 2014). Being anticancer agent, it suppresses the proliferation, migration, and invasion stages and mediates the miR‐34a upregulation through p53, and downregulates the Rac1 expression. Thymoquinone regulates pro‐ and antiapoptotic genes, and reduces the NF‐κB and IKKα/β phosphorylation and ERK1/2 and PI3K activities (Imran et al., 2018).

Uncoupling protein‐1 (UCP‐1) is the index protein of the brown adipose tissue. In recent study conducted by group of researchers, they found that thymoquinone treatment to the experimental subjects, which significantly increased the serum total antioxidant capacity, caused reduction in waist circumference, body weight, and body mass index (Mousavi et al., 2018; Namazi et al., 2018; Tüfek et al., 2015).

Glucagon‐like peptide‐1 (GLP‐1) analogs have been found to improve the glycemic control, while administration of streptozotocin is used to induce the diabetes in experimental subjects. In diabetic rats, supplementation of thymoquinone by using oral route in dose‐dependent manner exhibited elevation in plasma GLP‐1 levels by lowering the hypergaphy (Harphoush et al., 2019; Lee et al., 2019; Lee, Kim, et al., 2019). In diet‐induced obesity mouse model, supplemented thymoquinone at the rate of 20 mg/kg BW per day prevented diabetic phenotype via lowering the glucose concentration, fasting insulins, serum cholesterol, triglycerides, and inflammatory markers resistin and MCP‐1, improving the glucose tolerance and insulin sensitivity, and enhancing the phosphorylated Akt level, whereas it phosphorylated SIRT‐1 in skeletal muscle and phosphorylated SIRT‐1 and AMPKα in liver (Shpetim et al., 2017).

## 2 HEALTH PERSPECTIVES

### 2.1 Cancer insurgence

**FIGURE 1:**
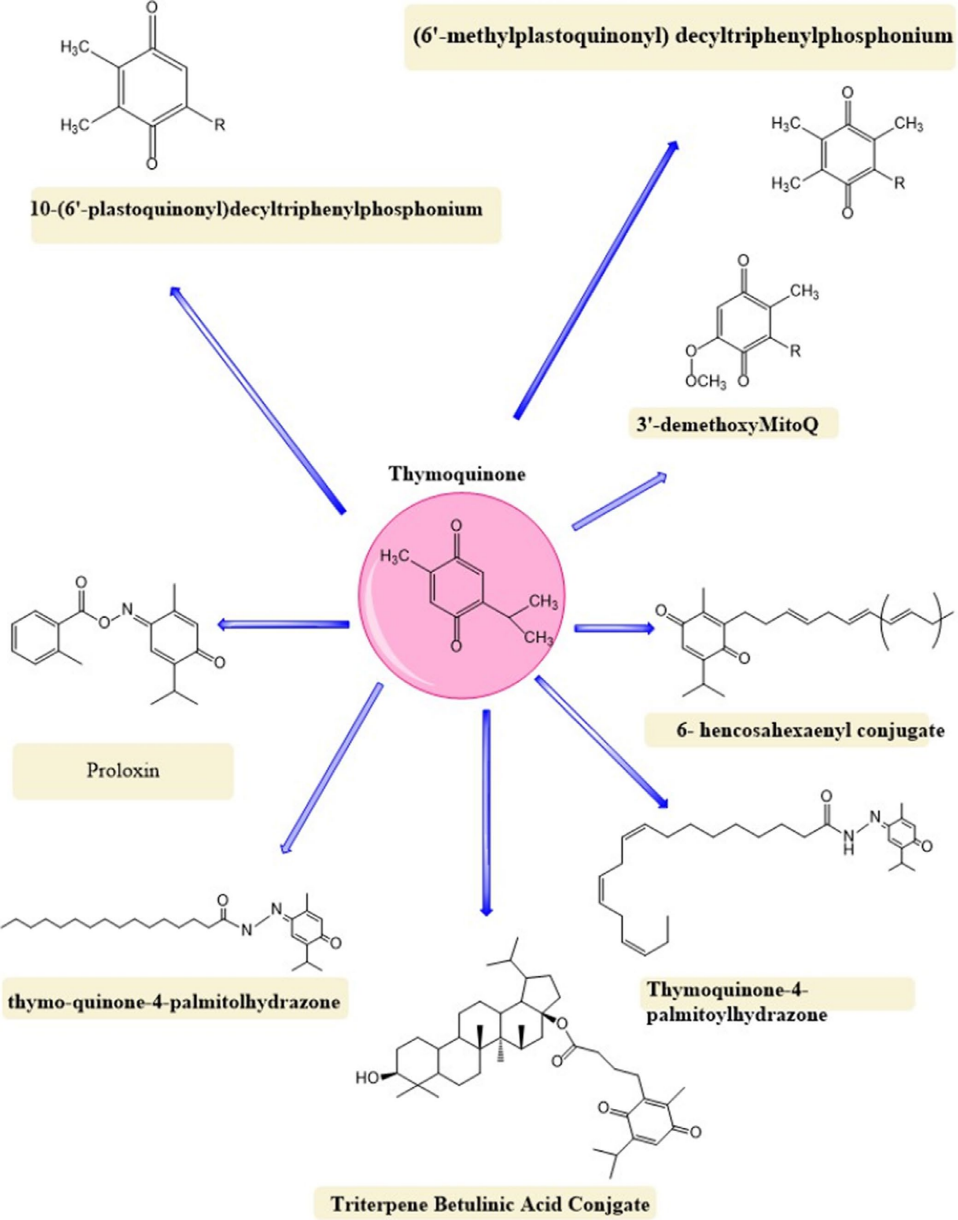
Thymoquinone and its derivatives

**FIGURE 2:**
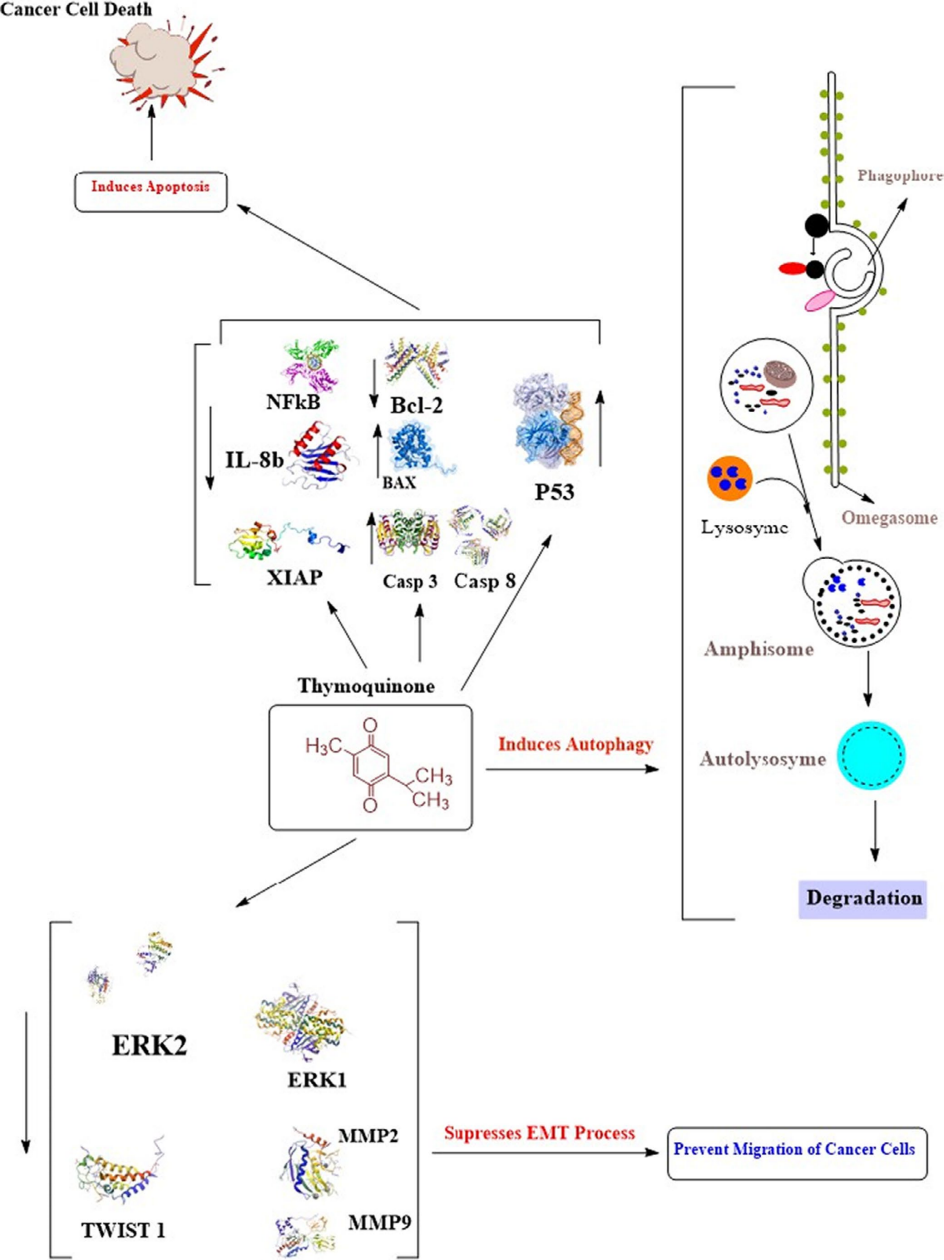
Scheme of anticancer potential of thymoquinone (modified from Khan et al., 2017)

Thymoquinone being potent anticancer agent significantly lowers the hypoxia‐inducible factor‐1α (HIF‐1α) expression, inhibits interaction between HSP90 and HIF‐1α, and boosts HIF‐1α protein degradation in hypoxic cancer cells. It also suppresses the downstream genes of HIF‐1α, exhibited alterations in concentrations of lactate, glucose, and ATP levels, which further linked with disturbance of anaerobic metabolic and induction of apoptosis (Lee, Kuo, et al., 2019; Lee, Kim, et al., 2019). Thymoquinone also suppresses the cancer cell stages and the activation of PI3K/Akt pathway in oral squamous cell carcinoma (Ren & Luo, 2019). Moreover, thymoquinone administration in AGS cell lines also lowers the cell propagation rate and causes the induction of apoptotic cell death, and downregulates the VEGF‐A gene expression (Rashid et al., 2019). In recent silico docking study by Sumathi and their coworkers, apoptotic targets such as MDM2, Trail‐R, Bak, Bax, Bcl‐2, and DNA repair target PARP were effectively docked by thymoquinone treatment. The docking with PARP induced cell death, exhibited cell cycle arrest in the late apoptotic stage, and induced DNA damage. In addition, downregulation of PARP gene expression was also reported after thymoquinone treatment (Sumathi et al., 2019). In another investigation, Ndreshkjana et al. (2019) summarized the anticancer effects of thymoquinone in combination with 5‐fluorouracil (5‐FU) against colorectal cancer cell lines via various mechanisms such as (a) deregulation of gene expression, (b) elimination of CD133 + CSC population, (c) downregulation of PI3K/AKT and WNT/ß‐catenin pathways, (d) eradication of propagated 3D tumor cell spheres at subtoxic doses, (e) inhibition of cell adhesion, and (f) reduction of transcriptional activity of ß‐catenin, respectively (Ndreshkjana et al., 2019). Thymoquinone also downregulates the expressions of containing plant homeodomain (PHD) and really interesting new gene (RING) finger domains 1 *DNMT1,3A,3B*, (*UHRF1*), *HDAC1,4,9, G9A, KMT2A,B,C,D,E*, and *KDM1B* genes in Jurkat cells and MDA‐MB‐468 cancer cell lines (Qadi et al., 2019). Multiple researchers and investigators determined the potent role of thymoquinone in combination with cyclophosphamide by applying different concentrations (0.5 mM‐10 μM) against Her2 + breast cancer cells through inhibiting the proliferation through the accumulation of cells in sub‐G1 (5.49%) and G1 (57.72%), whereas 12% cells were shifted from G2/M phase. On other side, combination of both compounds (0.5 mM‐20 μM) showed 16.6% of arresting cells in sub‐G1 and only 3.54% cells were remained in G2/M phase. Nonetheless, alleviation in PI3K/Akt signaling pathway via upregulating the PTEN and downregulating the Akt phosphorylation, and also reduction in expression of cyclin D1 were observed after both compounds’ administration in Her‐2 cells (Aumeeruddy & Mahomoodally, 2019; Bimonte et al., 2019; Khan et al., 2019). In different human cancer cell lines (T98 and LnCaP) and mouse embryonic fibroblast cell lines (3T3), thymoquinone dose‐dependently lowered the cell numbers and induced apoptosis via activating the caspase‐9 (Kus et al., 2018). A study described by the Subburayan and their colleagues found that human glioma cells treated with thymoquinone showed inhibition in cell growth via inducing Par‐4 expression, which triggers cellular senescence and prostate apoptosis response‐4 (Par‐4) tumor suppressor protein expressions. Enhancement in cellular size, G1 phase arrest, β‐galactosidase staining, and expression of senescence markers (p21, p53, Rb), and reduction in cyclin E, lamin B1, and cyclin depended kinase‐2 (CDK‐2) were reported after thymoquinone treatment (Table 1; Figures 1 and 2).

**Table 1:** Health‐endorsing perspectives of thymoquinone

Disorders	Mechanisms	References
Anticancer	Lowered the hypoxia‐inducible factor‐1α (HIF‐1α) expression, suppressed the interaction between HSP90 and HIF‐1α, and inhibited the downstream genes of HIF‐1α Altered the lactate, glucose, and ATP levels	Lee, Kuo, et al. (2019), Lee, Kim, et al. (2019)
	Suppressed the activation of PI3K/Akt pathway	Ren and Luo (2019)
	Decreased the cell propagation rate and induced apoptotic cell death Downregulated the VEGF‐A gene expression	Rashid et al. (2019)
	Docked apoptotic targets such as Bak, Bax, MDM2, Bcl‐2, Trail‐R, and DNA repair target PARP Exhibited cell cycle arrest in the late apoptotic stage Downregulated the PARP gene expression	Sumathi et al. (2019)
	Alleviated PI3K/Akt signaling pathway Upregulated the PTEN expression Downregulated the Akt phosphorylation Lowered the expression of cyclin D1	Aumeeruddy and Mahomoodally (2019), Bimonte et al. (2019), Khan et al. (2019)
	Reduced the cell numbers and induced apoptosis via activating the caspase‐9	Kus et al. (2018)
	Lowered the vascular endothelial growth factor‐A, cell viability, messenger RNA expression levels of human telomerase reverse transcriptase, nuclear factor kappa B genes Increased the tensin homolog and cyclin‐dependent kinase inhibitor 1 (p21) mRNA expression levels and active CASP‐3 protein level	Ozturk et al. (2018)
Anti‐diabetic	Lowered the concentrations of the triglycerides, low‐density lipoprotein, cholesterol Enhanced high‐density lipoprotein, glucose‐induced insulin secretion, and insulin sensitivity	Pelegrin et al. (2019)
	Improved the vasorelaxant responses of aortic rings to Ach. Increased eNOS in mRNA expression level and function, but lowered VCAM‐1 and LOX‐1 expressions	Abbasnezhad et al. (2019)
	Lowered fasting plasma glucose and glycemic status	Askari et al. (2019)
	Lowered blood glucose level and increased insulin levels, catalase, and GSH activities Improved the histopathological picture and hepatic glycogen contents	Abdelrazek et al. (2018)
	Lowered glucose concentrations and glycated hemoglobin	Rani et al. (2018)
Oxidative stress	Reduced the concentrations of alanine transaminase, total bilirubin, aspartate transaminase, and total protein Decreased the expression of iNOS and caspase 3 proteins Enhanced the thioredoxin protein expression	Atteya et al. (2015)
	Lowered the butyrylcholinesterase, alkaline phosphatase concentrations, and lipid peroxidation Enhanced the total antioxidant capacity and total thiol molecule	Hassanein and El‐Amir (2018), Nili‐Ahmadabadi et al. (2018)
	Lowered nitric oxide, p53, and Bax expressions Increased Bcl2 mRNA expression (heart tissue) and total antioxidant capacity	Jalili et al. (2018)
	Enhanced the concentrations of antioxidant enzymes such as superoxide dismutase and glutathione peroxidase Increased activation of Nrf2/heme oxygenase 1 (HO‐1) signaling pathway	Hu et al. (2019)
Antiobesity	Elevated plasma GLP‐1 levels by lowering the hypergaphy	Harphoush et al. (2019), Lee, Kuo, et al. (2019), Lee, Kim, et al. (2019)
Anti‐inflammatory	Reduced the escape latency time and the time spent in the target quadrant Lowered the mRNA expression of IL‐1β, IL‐6, monocyte chemoattractant protein‐1, and cyclooxygenase‐2	Guan et al. (2018)
	Lowered serum IL‐1β level and oxidative stress index, and enhanced the total antioxidant capacity.	Dur et al. (2016)
	Lowered the nuclear factor kappa‐light‐chain‐enhancer of activated B cells, inducible nitric oxide synthase expressions, and tumor necrosis factor‐alpha levels Enhanced the concentrations of glutathione peroxidase activity, glutathione peroxidase activity, and total antioxidant status	Zeren et al. (2016)
Cardioprotective	Reduced the infarct size, cardiac lactate dehydrogenase (LDH), and creatine kinase‐MB (CK‐MB) levels	Xiao et al. (2018)
	Enhanced the levels of p53 and Bax	Sezen et al. (2018)
	Lowered the congestion, edema, and pycnotic nuclei Increased the expression of antiapoptotic protein Bcl‐2	Adalı et al. (2016)
	Inhibited angiotensin II (Ang II)‐induced VSMCs' cell cycle progression, cyclin D1 expression Altered p21 expression and reduced MMP‐9 expression, ROS production, and NADPH oxidase activity Restored Ang II‐inhibited expression of p‐AMPK, PPARγ, and peroxisome proliferator‐activated receptor‐γ coactivator‐1α (PGC‐1α) proteins	Pei et al. (2016).
Hepatoprotective	Prevented from the elevation in liver enzymes Enhanced the concentrations of superoxide dismutase levels and ameliorated the histopathological alterations	Noorbakhsh et al. (2018), Tekbas et al. (2018), Zeinvand‐Lorestani et al. (2018)
	Lowered the concentrations of AST, ALT, ALP, and TBARs.	Sayeed et al. (2017)
	Decreased the myeloperoxidase (MPO) activities, malondialdehyde (MDA) level, and NO production Upregulated eNOS and downregulated iNOS and NOSTRIN expressions	Abd‐Elbaset et al. (2017)
	Enhanced total antioxidant capacity, reduced hepatic TNF‐α, increased IL‐10, lowered BAX protein, and enhanced Bcl /expression	Awad et al. (2016)
Neuroprotective	Enhanced the expression of 4 antioxidant, neuroprotective proteins: biliverdin reductase A, glutaredoxin‐3, mitochondrial ion protease, and 3‐mercaptopyruvate sulfurtransferase Decreased the expression of inflammatory cytokines, IL‐6, IL‐2, IL‐4, IL‐10, and IL‐17a Downregulated the chemokine (CC motif) ligand 3 (CXCL3), chemokine (CC) motif ligand 5 (CCL5), and complement factor B (CFB)	Cobourne‐Duval et al. (2018)
	Induced apoptotic cell death and Aβ formation	Cascella et al. (2018), Farkhondeh et al. (2018), Fouad et al. (2018)
	Decreased intracellular ROS generation, mitochondrial dysfunction, and apoptotic events Lowered mitochondrial membrane potential (Δψm)	Firdaus et al. (2018)
	Prevented rotenone‐induced motor defects and changes in the Parkin, dynamin‐related protein‐1 (Drp1), dopamine, and TH levels in the substantia nigra (SN) and striatum (ST) of dopaminergic areas	Ebrahimi et al. (2017)
Reproductive	Reproductive toxicity of male rats induced by cadmium chloride (CdCl_2_) but ameliorated the deleterious effects of CdCl_2_ probably by activating testicular endocrine and antioxidant systems	Parhizkar et al. (2016), Sayed et al. (2014)
	Improved sperm quality, testicular histology and oxidative/antioxidative status, and serum levels of LH, testosterone, and E2.	Hassan et al. (2019)
	Lowered the nitric oxide level, enhanced the motility (total motility and progressive motility), germinal thickness, morphology, count, viability of sperm cells, and testosterone hormone	Miah et al. (2018), Salahshoor et al. (2018)

Further, overexpression of Par‐4 significantly increases the expression of p53 and its downstream target p21, and increases β‐galactosidase‐positive cells, while siRNA/shRNA‐mediated knockdown of Par‐4 reverses the thymoquinone‐induced effects (Subburayan et al., 2018). In glioblastoma cells, thymoquinone also significantly suppresses the tumorigenic processes through proliferation, invasion, migration, counteract carcinogenesis, and angiogenesis (Chowdhury et al., 2018; Gruber et al., 2018; Johnson‐Ajinwo et al., 2018). Anticancer effects are improved when it was combined with genistein bioactive compound against thyroid cancer cells. Both compounds momentously lowered the vascular endothelial growth factor‐A, cell viability, messenger RNA expression levels of human telomerase reverse transcriptase, and nuclear factor kappa B genes, and also enhanced the tensin homolog and cyclin‐dependent kinase inhibitor 1 (p21) mRNA expression levels and active CASP‐3 protein level (Ozturk et al., 2018).

Hatiboglu and their colleagues explored the in vitro cytotoxic effect of thymoquinone dose‐dependently on B16‐F10 melanoma cell of mice, and intracerebral melanoma in vivo enhanced the cytotoxicity through inducing the apoptosis and DNA damage, enhancing the intracellular reactive oxygen species, suppressing the p‐STAT3, and regulating the proapoptotic and antiapoptotic proteins (Hatiboglu et al., 2018). The significant and momentous enhancement in hippocampal neurons is linked with increment in the doublecortin expression on both gene and protein levels, and reduction in caspase‐3 expression and the cleavage of poly‐ADP ribose polymerase, which were observed after thymoquinone treatment. On other side, thymoquinone has not shown any affect on gene expression of synaptophysin, synapsin, AKT, NGF, NF‐kB, p53, and Bax, and protein expression of nNOS and BDNF (Beker et al., 2018). A study reported by Diab‐Assaf et al. (2018) supplemented thymoquinone in HTLV‐1‐negative (CEM and Jurkat) malignant T lymphocytes and HTLV‐1‐positive (C91‐PL and HuT‐102) malignant T lymphocytes, which prevented the proliferation, induced apoptosis, enhanced the DNA fragmentation, decreased Bcl‐2α and TGF‐α expressions, and enhanced p53, TGF‐β1, and p21 levels (Diab‐Assaf et al., 2018).

A peer group of researchers and investigators investigated the effect of thymoquinone with different doses (2 and 4 mg/kg) dose‐dependently against MDA‐MB‐231 triple negative breast cancer in experimental mouse via inhibiting CXCR4 expression, tumor growth, and tumor vascularity along with suppressing the brain, lung, and bone metastases. Moreover, thymoquinone also suppresses the NF‐κB binding to the CXCR4 promoter and downregulation of the nuclear factor kappa‐light‐chain‐enhancer of activated B‐cell (NF‐κB) activation (Ahmad et al., 2018; Shanmugam et al., 2018). Breast cancer cells lines (MDA‐MB‐231 and MCF‐7) treated with thymoquinone to experimental subjects exhibited cell cycle arrest at sub‐G1 phase, and induced cell death (Kommineni et al., 2019). A peer group of researchers (Ekinci et al., 2018; Samarghandian et al., 2019) in another study evaluated the anticancer role of thymoquinone against human lung cancer cell line (A549 cells) via lowering the viability, inducing apoptotic cell death, enhancing the Bax/Bcl‐2 ratio, upregulating the p53 expression, and activating the caspase‐3 and caspase‐9, respectively (Ekinci et al., 2018; Samarghandian et al., 2019). Similarly, thymoquinone significantly upregulated the p53, downregulated the Bcl2, and induced apoptosis in MCF‐7 cells (El‐Far et al., 2018). Haron and their coworkers in another study investigated that thymoquinone suppressed the growth of Hep3B at IC50 < 16.7 μM for 72 hr, induced cell cycle arrest at the G1 checkpoint and non‐phase‐specific cell cycle arrest, and activated the caspases‐3/7 (Haron et al., 2018). Temozolomide in combination with thymoquinone has synergistic cytotoxic effect on U87MG cells through lowering the cell invasion and matrix metalloproteinases 2, 9 (Pazhouhi et al., 2018). In addition, clinical data have been reported on in vitro studies to explore the role of thymoquinone as an anticancer agent. In vitro, thymoquinone in renal cell cancer lines (ACHN &786‐O) of xenograft model markedly suppressed the metastatic capacity through AMPK/mTOR signaling pathway, inhibited the migration and invasion, induced autophagy, and suppressed the EMT dose‐dependently (Zhang et al., 2018).

## 3 ANTIDIABETIC ROLE

In recent study, thymoquinone has been found as potent antidiabetic agent in healthy male volunteers via decreasing the concentrations of the cholesterol and triglycerides, and enhancing the high‐density lipoprotein, glucose‐induced insulin secretion, and insulin sensitivity (Pelegrin et al., 2019). Daily administration of Nigella seed extracts with diverse doses, that is, 100, 200, and 400 mg/kg, by gavage to streptozotocin‐induced diabetic rat caused improvements in atherogenic index and reductions in serum glucose and lipids. Improvement in vasorelaxant responses, reduction in VCAM‐1 and LOX‐1 expressions (vascular cells of aortic tissue), and enhancement in eNOS in mRNA expression level and function were reported after thymoquinone treatment (Abbasnezhad et al., 2019). Thymoquinone markedly decreased the fasting plasma glucose (FPG) and glycemic status in experimental volunteers (Askari et al., 2019). In another investigation, it has been reported that thymoquinone significantly prevented male Wistar experimental rats from streptozotocin‐induced diabetes via lowering blood glucose level, and increasing insulin levels, and catalase and GSH activities. Furthermore, thymoquinone also enhanced the mean pancreatic islet diameter, and percentage of insulin immunoreactive parts, which improved the histopathological picture and hepatic glycogen contents (Abdelrazek et al., 2018). Safhi et al. (2019) investigated the effect of thymoquinone alone and combination of thymoquinone + fluoxetine in depressive type 2 diabetic rats. Similarly, momentous reduction in immobility time, increased latency to immobility, glucose concentrations, enhancement in locomotor activity, and reduction in antioxidant enzymes, TBARS, and restoration of GSH activities were reported after thymoquinone treatment. Thymoquinone in combination with fluoxetine considerably decreased the inflammatory marker (IL‐6, IL‐1β, and TNF‐α) levels (Safhi et al., 2019). It also has been established that different doses of thymoquinone, that is, 20, 40, and 80 mg/kg metformin (150 mg/kg), and their nanoformulations (20, 40, and 80 mg/kg for thymoquinone and 80 mg/kg for metformin) induced to diabetic experimental rats lowered glucose concentrations and glycated hemoglobin dose‐dependently (Rani et al., 2018). Atta et al. (2018) determined that thymoquinone at 50 mg/kg BW for 12 weeks administrated to diabetic Wistar male rats induced by intraperitoneal infusion of streptozotocin, 65 mg/kg through stomach gavage, prevented them from the diabetes‐caused cardiac complications via declining the plasma nitric oxide level and enhancing superoxide dismutase antioxidant enzyme. It also downregulated the expression of cardiac‐inducible nitric oxide synthase, and upregulated the erythropoietin genes, nuclear factor‐erythroid‐2‐related factor 2 (Nrf2) protein, and vascular endothelial growth factor. It also showed inhibition on C‐reactive protein, E‐selectin level, and interleukin‐6 along with normalized plasma cardiac markers (creatine kinase and troponin), respectively (Atta et al., 2018; Ozer et al., 2018). Likewise, thymoquinone also prevented mature male Wistar rats from streptozotocin‐induced diabetes via decreasing blood glucose concentrations and enhancing the insulin level (Abdelrazek et al., 2018). In another investigation performed by Safhi et al. (2019), significant reduction in blood glucose level, TBARS level, inflammatory marker (IL‐1β, IL‐6, and TNF‐α) levels, and immobility time, and enhancement in concentrations of antioxidant enzymes, latency to immobility, and locomotor activity were reported in depressive type 2 diabetic rats after thymoquinone treatment (Safhi et al., 2019). Different researchers and investigators explored the potential role of thymoquinone, metformin, and their nanoformulations against the streptozotocin–nicotinamide‐induced diabetic rats by applying different doses (20, 40, and 80 mg/kg), (150 mg/kg) and their nanoformulations (20, 40, and 80 mg/kg for thymoquinone and 80 mg/kg for metformin) via lowering glucose level and glycated hemoglobin (Negi et al., 2018; Rani et al., 2018). Multiple mechanisms are involved in antidiabetic role of thymoquinone (30 mg kg^−1^ day^−1^) against experimental subjects such as reduction in levels of glucose, glycosylated hemoglobin levels, urea and creatinine, NF‐κB levels, and liver enzyme concentrations (aspartate aminotransferase, alanine aminotransferase, and gamma‐glutamyl transpeptidase activities), respectively (Usta & Dede, 2017). In another findings, obesity was induced through diet and enhanced the blood glucose and insulin levels, decreased the insulin sensitivity, enhanced the cholesterol and triglyceride concentrations, lowered the protein expression of phosphorylated Akt, and increased NADH/NAD + ratio and serum levels of inflammatory markers MCP‐1, and resistin. Obesity is further accompanied by reduction in phosphorylated SIRT‐1 in skeletal muscle, phosphorylated SIRT‐1, and AMPKα in liver. Supplementation of thymoquinone (20 mg kg^−1^ bw^−1^ day^−1^) bioactive compounds to obese rats reverted these changes in the experimental rats (Karandrea et al., 2017). In adult male Wistar rats, diabetes was induced by streptozotocin, while orally supplemented thymoquinone (35 mg kg^−1^ day^−1^) to the experimental subjects exhibited improvement in the glucose–insulin homeostasis‐related parameters, lipid profile parameters, integrity of pancreatic islets, and hepato‐renal functional and histomorphological statuses and enhanced the upregulated survivin, insulin‐producing β cells, endothelial cluster of differentiation 31, vascular endothelial growth factor, total glutathione, interleukin‐10 (IL‐10), and superoxide dismutase. Additionally, thymoquinone in the pancreatic tissues of STZ diabetic rats also downregulates the IL‐1β, caspase‐3, and TBARS levels (El‐Shemi et al., 2018).

### 3.1 Oxidative stress

Acetaminophen (APAP) has been found to induce hepatotoxicity via depleting the concentration of glutathione enzyme, which further led to lipid peroxidation and subsequent liver injury. Thymoquinone in combination with curcumin prevented rats from liver injury through lowering the concentrations of alanine transaminase, total bilirubin, aspartate transaminase, and total protein. Additionally, reduction in iNOS and caspase 3 protein expressions and increment in the thioredoxin protein expression were also reported (Atteya et al., 2015). Intraperitoneal administration of diazinon (16 mg/kg) subjected to experimental subjects significantly showed enhancement in concentration of butyrylcholinesterase, alkaline phosphatase, alanine aminotransferase, nitric oxide, aspartate aminotransferase, lipid peroxidation, and (ALP), and also depleted the total antioxidant capacity and total thiol molecule (Nili‐Ahmadabadi et al., 2018). In human retinal pigment epithelium (RPE) cells, induction of oxidative stress by hydrogen peroxide promotes the age‐related macular degeneration, whereas administration of thymoquinone was found to improve the cell viability, induce apoptosis, lower reactive oxygen species and malondialdehyde, and also increase the concentrations of glutathione peroxidase and superoxide dismutase along with enhancement in activation of Nrf2/heme oxygenase 1 (HO‐1) signaling pathway (Hu et al., 2019) . Thymoquinone works as potent antioxidant via decreasing the concentration of production of peroxides, alanine aminotransferase, and aspartate aminotransferase enzymes (Hassanein & El‐Amir, 2018) . Jalili and their coworkers in previous investigations found that thymoquinone in combination with morphine treated with experimental animals markedly caused reduction in nitric oxide, p53, and Bax expressions, and enhancement in Bcl2 mRNA expression (heart tissue) and total antioxidant capacity (Jalili et al., 2018).

In experimental subjects, intraperitoneal administration of thymoquinone at the rate of 10 mg/kg significantly prevented those from the oxidative damage via enhancing the levels of antioxidant enzymes such as superoxide dismutase and catalase activity (Zeinvand‐Lorestani et al., 2018). Similarly, fipronil (10 mg/kg bw) treated with male Wistar rats considerably increased the concentration of aspartate transferase, γ‐glutamyl transferase, uric acid, urea, creatinine, lactate dehydrogenase, alanine transferase, and alkaline phosphatase, as well as also reduced the glutathione peroxidase, superoxide dismutase, and catalase enzymes in the renal, hepatic, and brain tissues. On other side, thymoquinone (10 mg/kg bw) supplied to these rats reverted these changes (Abdel‐Daim et al., 2018). Cardiotoxicity induced by intraperitoneal administration of doxorubicin (15 mg/kg) enhanced the creatinine kinase‐MB, lactate dehydrogenase, and aspartate aminotransferase, whereas orally administrated thymoquinone with different doses at 10 and 20 mg/kg BW caused momentous reductions in lactate dehydrogenase, creatinine kinase‐MB, aspartate aminotransferase, and inflammatory cytokine (IL‐2), as well as also increased the antioxidant enzyme concentrations (Alam et al., 2018). Multiple investigations by different researchers also determined hepatoprotective role of thymoquinone in male Wistar rats. They investigated that thymoquinone (30 mg/kg) to male Wistar rats suppressed the expression of apoptotic effectors, lowered the ALT level, and attenuated endoplasmic reticulum stress parameters (Bouhlel et al., 2018; Cascella et al., 2018). Intraperitoneally supplemented aluminum trichloride (10 mg kg^−1^ day^−1^) and D‐galactose (60 mg kg^−1^ day^−1^) to experimental rats induced neurobehavioral and neuropathological alterations, whereas intragastrically administrated thymoquinone (20 mg kg^−1^ day^−1^) improved cognition, increased antioxidant enzymes level and B‐cell lymphoma‐2 levels, and decreased the acetylcholinesterase activities and nitric oxide in whole brain (Abulfadl et al., 2018). A study described by Chen and their followers explored the preventive role of thymoquinone by applying the intragastric administration (30 mg/kg) against spinal cord injury in rats through enhancing the Basso, Bresnahan, and Beattie score, lowering the water contents, tumor necrosis factor α, IL‐6 and IL‐18, interleukin (IL)‐1β, and oxidative stress, inhibiting the COX‐2 protein expression and prostaglandin E2 activity, and activating the PI3K, PPAR‐γ, and p‐Akt protein expression (Chen et al., 2018).

During partial hepatectomy, thymoquinone at the rate of 30 mg/kg treated with rats prevented those from ischemia/reperfusion via reducing the alanine aminotransferase, increasing antioxidant enzymes, attenuating the endoplasmic reticulum stress parameters, and repressing the expression of apoptotic effectors along with improving the mitochondrial function (Bouhlel et al., 2018). Similarly, Meral has determined that intraperitoneal (i.p.) injection of thymoquinone at 10 mg/kg momentously caused reductions in expression of miR‐206b‐3p, oxidative stress, and necrosis in the liver tissue in Ehrlich acid solid tumor model‐induced male BALB/c mice (Meral et al., 2018). Atorvastatin is used to induce hepatic injury in male Sprague Dawley rats, which is linked with reduction in liver enzymes, protein carbonylation, malondialdehyde lipid peroxidation marker, and caspase 3 activity enhancement in reduced glutathione and catalase (Hassan et al., 2018). Supplementation of doxorubicin (15 mg/kg, i.p.) enhanced the serum enzyme marker, that is, creatinine kinase‐MB, lactate dehydrogenase, and caused enhancement in oxidative stress marker lipid peroxidation, aspartate aminotransferase along with reductions in antioxidant enzymes, and enhancement in inflammatory cytokine (IL‐2) while thymoquinone (20 mg/kg b/w, p.o.) reverted these changes (Alam et al., 2018).

## 4 ANTI‐INFLAMMATORY ROLE

Cerebral small vessel disease is covering a variety of abnormality‐related small blood vessels that degrade the cognition, which further leads to stroke. Thymoquinone phytochemical is working as an anti‐inflammatory agent and prevented spontaneous hypertensive rats from the cerebral small vessel disease through various mechanisms such as reduction in systolic blood pressure, escape latency time, and the time spent. It also significantly increased along with momentous reduction in mRNA expression of IL‐6, monocyte chemoattractant protein‐1, IL‐1β, and cyclooxygenase‐2 in brain of spontaneous hypertensive rats. Moreover, thymoquinone significantly enhanced the concentrations of antioxidant enzymes, and lowered the MDA level (Guan et al., 2018). Thymoquinone has anti‐inflammatory effects on cerulein‐induced acute pancreatitis of male Wistar albino rats via decreasing serum IL‐1β level and oxidative stress index, and enhancing the total antioxidant capacity (Dur et al., 2016). Induction of acetylsalicylic acid to male Wistar Albino rats caused gastric ulcers and enhanced the inducible nitric oxide synthase expressions, nuclear factor kappa‐light‐chain‐enhancer of activated B cells, and tumor necrosis factor‐alpha levels along with reductions in antioxidant enzymes. On other side, administration of thymoquinone to experimental subjects reverted these changes (Zeren et al., 2016). A group of researchers and investigators (Amin & Hosseinzadeh, 2016; Boudiaf et al., 2016) found that thymoquinone exhibited suppression in the fMLF‐induced superoxide production and granule exocytosis in neutrophils, attenuation in specific and azurophilic granule exocytosis in fMLF‐stimulated neutrophils, reduction in cell surface expression of gp91(PHOX) and CD11b, impaired the phosphorylation on Ser‐304 and Ser‐328 of p47(PHOX), and release of myeloperoxidase (Amin & Hosseinzadeh, 2016; Boudiaf et al., 2016). A study found that thymoquinone treated with lipopolysaccharide (LPS)‐stimulated BV‐2 murine microglia cells lowered NO2(−) with an IC50 of 5.04 μM, pro‐inflammatory cytokines IL‐6, IL‐12p40/70, CCL2/MCP‐1, CCL12/MCP‐5, and G‐CSF, and attenuated MCP‐5 and MCP‐1 protein (10 μM) (Taka et al., 2015). During macrophage process, TNF‐α promotes the rheumatoid arthritis while supplementation of thymoquinone (1‐5 μM) significantly suppressed the IL‐8 production, TNF‐α‐induced IL‐6 and VCAM‐1, ICAM‐1, and cadherin‐11 (Cad‐11) expression. Further, it also suppresses the phospho‐JNK expression, TNF‐α‐induced phospho‐p38, and apoptosis‐regulated signaling kinase 1 (ASK1) (Faisal et al., 2015; Umar et al., 2015). Wang and their colleagues determined the preventive dose‐dependent role of thymoquinone against LPS‐stimulated BV2 microglial cells via inhibiting IL‐1β, TNF‐α, NO, and PGE2 production, and suppressing the NF‐κB activation, and PI3K and Akt phosphorylation (Wang et al., 2015). When thymoquinone is applied to pancreatitis male Wistar rats, it influenced the apoptosis‐associated speck‐like protein (ASC) complex of NOD‐like receptor pyrin domain‐containing 3 (NLRP3) expression, and received momentous reduction in the serum lipase (L)/amylase (A) ratio and oxidative stress, and enhancement in mRNA expression of IL‐18, IL‐1β, and TNF‐α in antioxidant enzymes. In addition, thymoquinone also lowered the upregulation of mRNA and the protein expression of ASC and caspase‐1 (Periyanayagam et al., 2015). Likewise, thymoquinone exerts anti‐inflammatory affect on acute bacterial prostatitis (ABP) induced by pseudomonas aeruginosa in experimental subjects via decreasing the concentrations of prostate tissue MDA and NO levels, and enhancing the levels of antioxidant enzymes (Alemi et al., 2013; Rifaioglu et al., 2013).

## 5 CARDIOVASCULAR ROLE

In Langendorff‐perfused rat hearts, thymoquinone has significant impact on myocardial ischemia/reperfusion (I/R) injury through lowering infarct size, creatine kinase‐MB levels, and cardiac lactate dehydrogenase, promoting autophagy, improving cardiac function, and suppressing enedoxidative stress and apoptosis (Xiao et al., 2018). Moreover, thymoquinone also prevents Wistar albino rats from the myocardial ischemia/reperfusion via enhancing the levels of p53 and Bax (Sezen et al., 2018). Adli and their coworkers investigated that supplementation of thymoquinone (40 mg kg^−1^ day^−1^) to adult male Wistar Albino rats prevented rats from the cisplatin (15 mg/kg dose)‐induced myocardial injury, which further caused reduction in edema, congestion, and pycnotic nuclei in myocardial fibers, and enhancement in antiapoptotic protein Bcl‐2 level (Adalı et al., 2016). Diabetes and their associated diseases are linked with the propagation of vascular smooth muscle cells, whereas thymoquinone dose‐dependently prevented rats from these complications through multiple mechanisms such as inhibition of cyclin D1 expression, angiotensin II (Ang II)‐induced VSMCs' cell cycle progression, alteration in p21 expression, reduction in MMP‐9 expression, ROS production, and NADPH oxidase activity, and enhancement in superoxide dismutase activity. Restoration of Ang II‐inhibited expression of p‐AMPK, peroxisome proliferator‐activated receptor‐γ coactivator‐1α proteins, and PPARγ were reported dose‐dependently after thymoquinone treatment (Pei et al., 2016). Detremmerie et al. (2016) investigated the role of thymoquinone in isolated arteries by causing the endothelium‐dependent augmentation of contractions and augmenting the production of cIMP (Detremmerie et al., 2016). Thymoquinone also prevented Wistar rats induced by isoproterenol (125 mg/kg) by applying the different oral doses such as 12.5, 25, and 50 mg/kg through multiple mechanisms such as enhancement in SOD and myocardial ratio and reduction in lactate dehydrogenase and thiobarbituric acid levels from the myocardial injury (Ahmad & Beg, 2013; Randhawa et al., 2013; Tufail et al., 2020). Cyclophosphamide is used to induce cardiotoxicity in experimental subjects by enhancing the concentrations of serum lactate dehydrogenase, urea, cholesterol, triglycerides, creatine kinase, creatinine, tumor necrosis factor‐α, total nitrate, and thiobarbituric acid reactive substances. It also reduced the glutathione, glutathione peroxidase, catalase, and adenosine triphosphate levels. On other side, thymoquinone at the rate of 50 mg/L in drinking water to experimental subjects reverted these changes (Nader et al., 2010; Nagi et al., 2011). Similarly, thymoquinone treated with rats with reperfusion injury showed momentous improvements in left ventricular function, attenuation in mitochondrial oxidative damage, reduction in myocardial infarct size and lactate dehydrogenase, enhancement in antioxidant enzymes, reduction in number of apoptotic cardiomyocytes, suppression of p53 acetylation, enhancement of mitochondrial function, upregulation of SIRT1 level, and reduction in production of H_2_O_2_, respectively (Lu et al., 2018). A study conducted by Gonca and Kurt showed that intraperitoneal supplementation of thymoquinone (10 mg/kg) to anesthetized rats prevented rats from the myocardial ischemia and ischemia‐ and reperfusion‐induced ventricular arrhythmias via multiple pathways such as reduction in infarct size, arrhythmia scores, and incidence of ventricular tachycardia and ventricular fibrillation (Gonca & Kurt, 2015).

The supplementation of thymoquinone at the rate of 10 mg kg^−1^ day^−1^ in the drinking water normalized the levels of SK(Ca), eNOS, the components of the angiotensin system, and IK(Ca), and restoration of EDHF‐mediated relaxations and NO− in the mesenteric artery of middle‐aged rats (Idris‐Khodja & Schini‐Kerth, 2012). Thymoquinone phytochemical is also capable of lowering the aortic MDA and attenuating the atherogenesis in a rabbit model of atherosclerosis (Ragheb et al., 2011). Thymoquinone also decreases the oxidized low‐density lipoprotein receptor‐1 (LOX‐1) gene, protein expression, macrophages and pro‐inflammatory cytokine level in apolipoprotein E knockout (ApoE^−/−^) male mice (Xu et al., 2018). In earlier study conducted by Lu and their colleagues, thymoquinone showed significant impact on rat hearts and neonatal rat (myocardial ischemia injury) caused momentous improvement in left ventricular function, reduction in myocardial infarct size, lactate dehydrogenase production, elevation of antioxidant enzymes, inhibition of p53 acetylation, enhancement of mitochondrial function, upregulation of SIRT1 expression, and reduction in number of apoptotic cardiomyocytes (Lu et al., 2018). Moreover, thymoquinone (0.2 ml/kg) in combination prevented experimental rats (Wistar albino) after myocardial ischemia from the lung injury and lowered the levels of p53 and Bax (Sezen et al., 2018).

## 6 HEPATOPROTECTIVE ROLE

Thymoquinone exerted protective effect on paraquat‐induced hepatotoxicity in adult male mice. Supplementation of 20 mg/kg thymoquinone prevented rats from the elevation in liver enzymes, enhanced the concentrations of superoxide dismutase levels, and ameliorated the histopathological alterations induced by paraquat (Noorbakhsh et al., 2018Tekbas et al., 2018; Zeinvand‐Lorestani et al., 2018). It also significantly lowers the concentrations of AST, ALT, ALP, and TBARs (Abd‐Elbaset et al., 2017; Sayeed et al., 2017). Likewise, thymoquinone formulated in liposome prevented experimental subjects from the cyclophosphamide‐induced liver toxicity and higher serum bilirubin concentration (Laskar et al., 2016). In nonalcoholic steatohepatitis (NAFLD) liver of rats, thymoquinone (10, 20 mg/kg) lowered the MDA level, enhanced total antioxidant capacity, reduced hepatic TNF‐α, increased IL10, lowered BAX protein, and enhanced Bcl expression (Awad et al., 2016). Elevation in liver enzymes such as aspartate aminotransferase, alanine aminotransferase, lactate dehydrogenase, total bilirubin, alkaline phosphatase, and gamma‐glutamyltransferase was reported in female rats after inducing the hepatotoxicity by tamoxifen. It also used to prevent rats from the lipid peroxidation and leakage of antioxidant enzymes, and enhance the tumor necrosis factor‐alpha in the liver, whereas thymoquinone supplementation (50 mg/kg/BW) reverted these changes (Suddek, 2014). Induction of carbon tetrachloride to experimental rats caused momentous elevation in alanine aminotransferase activity, reductions in glutathione concentrations, reductions in the NAD(P)H‐quinone oxidoreductase activities and messenger RNA (mRNA) levels of glutathione S‐transferase and microsomal epoxide hydrolase, and reduction in the glutathione and cysteine levels. On other side, thymoquinone application in corn oil (5 mg/kg) reverted these changes (El‐Sayed, 2011; Erdemli et al., 2018). It also enhances the antioxidant enzyme levels (superoxide dismutase, glutathione level, catalase, glutathione peroxidase, and glutathione reductase) in liver tissues in experimental subjects (Mabrouk, 2017). In Wistar rats, thymoquinone application (500 mg kg^−1^ day^−1^) provides protection against intraperitoneally administrated cisplatin (12 mg/kg/body weight)‐induced hepatotoxicity via enhancing the antioxidant enzyme activities, that is, reduced glutathione contents, and decreasing MDA, TNF‐α, iNOS, and IL‐1β (Al‐Malki & Sayed, 2014). Zafeer and their coworkers investigated the role of thymoquinone (10 μM) by providing the protection against the cadmium‐induced oxidative stress in the liver of Swiss albino rats through attenuating the protein oxidation and rejuvenating the depleted antioxidants of cellular fraction (Zafeer et al., 2012). Similarly, aflatoxin in experimental subjects also known to induce hepatotoxicity via enhancing the concentrations of liver enzymes, that is, ALT, ALP, and AST along with malondialdehyde levels, and reduce the glutathione concentrations, whereas thymoquinone administration to mouse reverted these changes (Nili‐Ahmadabadi et al., 2011). In addition, intraperitoneal supplementation of acetaminophen (500 mg/kg) significantly increased the concentrations of hepatic lipid peroxides, total nitrate/nitrite, and serum ALT, and lowered the hepatic reduced glutathione and adenosine triphosphate in a time‐dependent manner. On other side, different doses of thymoquinone (0.5, 1, and 2 mg kg day^−1^) to experimental mouse provide prevention against acetaminophen‐induced hepatotoxicity dose‐ and time‐dependently via lowering the serum alanine aminotransferase activities (Nagi et al., 2010).

## 7 NEUROPROTECTIVE ROLE

Traumatic brain injury and microglial activation are pathological markers that lead to several neural disorders, that is, Alzheimer's disease and Parkinson's disease. Higher concentrations of free radicals and pro‐inflammatory cytokines are released during the chronic activation of microglia. Thymoquinone (12.5 μM for 24 hr) phytochemical work as preventive agent when treated with interferon‐gamma (IFN‐γ)‐activated BV‐2 microglial cells and lipopolysaccharide (LPS) by momentously enhancing the expression of 4 antioxidant, neuroprotective proteins: glutaredoxin‐3, biliverdin reductase A, 3‐mercaptopyruvate sulfurtransferase, and mitochondrial ion protease, as well as also lowering the expression of inflammatory cytokines, IL‐6, IL‐2, IL‐4, IL‐10, and IL‐17a, respectively. Additionally, thymoquinone also downregulated the chemokine (CC) motif ligand 5, chemokine (CC motif) ligand 3, and complement factor B (CFB) (Cobourne‐Duval et al., 2018).

Thymoquinone has protective role on arsenic (10 mg/kg/body weight; p.o.)‐induced toxicity in hippocampi of Wistar rats. It momentously decreased the mitochondrial dysfunction, mitochondrial membrane potential (Δψm), intracellular ROS generation, and apoptotic events (Firdaus et al., 2018). Different researchers and investigators found that different doses of thymoquinone at the rate of 2.5 and 10 mg/kg in rats exhibited neuromodulatory effect via inducing apoptotic cell death and Aβ formation resulting from glutamate administration (Cascella et al., 2018; Farkhondeh et al., 2018; Fouad et al., 2018). Thymoquinone also prevents rats from the progression of Parkinson's disease induced by rotenone. The supplementation of thymoquinone at the rate of 7.5 and 15 mg kg day^−1^, po, in male Wistar rats prevented rotenone‐induced motor defects and caused changes in the dynamin‐related protein‐1, parkin, dopamine, and TH levels in the striatum and substantia nigra of dopaminergic areas (Ebrahimi et al., 2017). Similarly, encapsulated thymoquinone in polylactic coglycolic acid chitosan nanoparticles considerably enhanced the locomotor activity and grip strength and lowered the ischemia infarct volume in the middle cerebral artery‐occluded rats (Xiao et al., 2016). Ramachandran and Thangarajan (2016) investigated that effective role of solid lipid nanoparticles encapsulated thymoquinone (10 and 20 mg/kg) against 3‐nitropropionic acid‐induced huntington's disease animals via multiple mechanisms such as improving the muscle strength, movement, rigidity, and memory performances, attenuating the levels of NO, LPO, and protein carbonyls in 3‐NP‐induced animals, controlling the mitochondrial SDH inhibition, restoring the antioxidant defense system, and alleviating anticholinergic effect upon 3‐NP induction. Moreover, thymoquinone also plays effective role against 3‐NP toxicity by protecting the striatal structural microelements (Ramachandran & Thangarajan, 2016). In experimental rats, orally administrated thymoquinone (5 mg kg^−1^ day^−1^) enhanced the neuron density in contralateral hippocampal regions (CA1, CA2‐3, and CA4) and lowered malondialdehyde level (Gülşen et al., 2016). With acrylamide‐induced neurotoxicity in both in vitro and in vivo of male Wistar rats, thymoquinone dose‐dependently (2.5, 5, and 10 mg/kg IP) significantly decreased abnormalities and lowered the level of MDA in cerebral cortex (Mehri et al., 2014). Thymoquinone also rescued dopaminergic neurons and decreased the release of lactate dehydrogenase, increased the mitochondrial membrane potential, enhanced lysosomal degradation, and inhibited mitochondria‐mediated apoptotic cell death (Radad et al., 2015). Thymoquinone (10 mg/kg) supplemented to male Wistar albino rats with spinal cord injury (SCI) lowered the histological features of spinal cord damage (Üstün et al., 2014). In animals, pentylenetetrazole (50 mg/kg) has been used to induce generalized seizures and mortality, prolong the onset of seizures, and lower the polyspike and epileptiform discharges and high‐grade seizures. It also caused reduction in calmodulin‐dependent protein kinase II (CaMKII), suppression in phosphorylation of cAMP response element‐binding protein (CREB) in cortex and hippocampus, and decline in gamma‐aminobutyric acid B1 receptor (GABAB1R) levels. It also enhanced the Bax, decreased Bcl‐2 expression, and activated caspase‐3. Thymoquinone in combination with vitamin C reversed these changes (Ullah et al., 2015). In unilateral intrastriatal 6‐hydroxydopamine (6‐OHDA)‐lesioned rats, diverse concentrations of thymoquinone (5 and 10 mg/kg BW) caused reduction in the number of neurons on the left side of the substantia nigra pars compacta, nitrite, and MDA level, and improved turning behavior (Sedaghat et al., 2014). In primary cultured cerebellar granule neurons, supplemented thymoquinone with different doses (0.1 and 1 μM) lessened the β‐amyloid peptide 1–40 sequence by preventing neurotoxic effects and neural cell death (Ismail et al., 2013). Multiple evidences and findings are reported by different researchers and investigators and found preventive role against Alzheimer's amyloid‐β peptide (Aβ)‐induced neurotoxicity in in vitro study in rat primary neurons. There are multiple pathways such as attenuation of Aβ1‐42‐induced neurotoxicity, suppression of mitochondrial membrane potential depolarization, inhibition of reactive oxygen species production, suppression of Aβ1‐42 aggregation, and restoration of synaptic vesicle recycling inhibition (Alhebshi et al., 2013; Kanter, 2011; Khan et al., 2012).

## 8 REPRODUCTIVE ROLE

Cadmium chloride is used to induce reproductive toxicity in male rats, while thymoquinone administration prevented the deleterious effects of cadmium chloride via activating testicular endocrine and antioxidant systems (Parhizkar et al., 2016; Sayed et al., 2014). Similarly, orally administrated lead (20 mg body weight) has been known to cause toxicity in male albino rats and lowered the sperm count, testis and epididymal weights, motility and viability, and serum FSH, LH, testosterone, and estradiol levels. It also lowered the testicular antioxidant molecules, caused enhancement in sperm abnormalities, downregulated the aromatase gene expression, activated the caspase‐3 apoptotic pathways, decreased the MDA and NO levels, and also exhibited the germinal epithelium sloughing, complete seminiferous tubules hyalinization, and hypocellularity. Moreover, thymoquinone treated with experimental rats reverted these changes (Hassan et al., 2019).

Moreover, morphine at the rate of 20 mg/kg applied to the experimental male mice caused momentous reductions in testis weight, germinal thickness, count, testosterone level, viability, morphology, and motility of sperm and enhancement in nitric oxide. On other side, different doses of thymoquinone (2, 10, and 20 mg/kg) and thymoquinone (2, 10, and 20 mg/kg) in combination with morphine (20 mg/kg) lowered the nitric oxide level, enhanced the motility (total motility and progressive motility), germinal thickness, morphology, count, viability of sperm cells, and testosterone hormone (Miah et al., 2018; Salahshoor et al., 2018). It also improved the sperm fertility rate and prevented anomalies. Moreover, thymoquinone treatment momentously enhanced the spermatogenic cells, mean volumes of testis, leydig cells, and seminiferous tubules (Tüfek et al., 2015). From another model, diabetic Wistar male rats induced by intraperitoneal injection of STZ (65 mg/kg) enhanced the malondialdehyde and nitric oxide level, upregulated the NF‐κB and nitric oxide, and lowered the antioxidant enzyme concentrations in testicles of diabetic rats, whereas oral‐administrated thymoquinone reverted these changes in subjects. Additionally, it also showed reductions in epididymal sperm count and improvement in low plasma testosterone level (Mabrouk & Ben Cheikh, 2016; Rathore et al., 2020).

### 8.1 Antiarthritic action

In adjuvant‐induced arthritis, thymoquinone treatment provides protection against rheumatoid arthritis via reducing the expressions of IL‐1b and TNF‐a in adjuvant‐induced arthritis (Vaillancourt et al., 2013). Moreover, ovalbumin‐induced asthma in mice displayed elevated levels of leukotrienes‐B4, C4, Th‐2 cytokines, and eosinophils in bronchoalveolar lavage fluid. TQ dealing perfected the pathological perturbations closely associated with airway inflammation by suppressing lipoxygenase (5‐LOX) and NF‐kb. El‐Gazzar et al. conveyed the initiation of suppressive NF‐kb homodimer obligatory to the promoter in LPS‐induced rat basophil cells, RBL‐2H3 (El Mezayen et al., 2011; El Gazzar et al., 2007). However, Sethi et al. observed that NF‐kb inhibition is attributed to additional TNF‐a‐induced Ik‐b degradation and phosphorylation along with p65 translocation (Sethi et al., 2008). Additionally, IL‐6 induced STAT3 phosphorylation in U266 multiple myeloma cells was found to be inhibited by TQ along with c‐Src and JAK‐2 activation. The study further revealed the interaction of cyclin D1, apoptotic proteins, surviving, Mcl‐1, and vascular endothelial growth factor in the U266 cells. TQ‐interceded decrease in peroxynitrite (NO–2) remained initiate in parallel with the weakening in iNOS protein manifestation. Further, the anti‐inflammatory activity of TQ was evaluated on 96 cytokines. TQ was found to diminish expression of Cxcl10 and different cytokines induced by LPS. TQ was also found to attenuate activated microglia and delay the onset of inflammation‐associated neurodegenerative diseases (Kodappully Sivaraman Siveen et al., 2014; Taka et al., 2015).

### 8.2 Effects of thymoquinone in respiratory diseases

Bronchial asthma is linked with airway inflammation and leukotrienes. Thymoquinone has been found to prevent from the deleterious effects induced by chemicals and environmental toxins. It also protects the lungs by exposing the toluene in rats (Kanter, 2011). Thymoquinone also prevents the deleterious effects of bleomycin on lung tissues of rats through lowering the pulmonary fibrosis development and activated NF‐kb overexpression, as well as corrected emphysema in inflammatory cell infiltration, air alveoli, and lymphoid hyperplastic cell initiation (El‐Khouly et al., 2012). It also works as an effective agent against cyclophosphamide‐persuaded pulmonary impairment in rats (El‐Khouly et al., 2012). Similarly, thymoquinone as bioactive compound has been found to reduce the levels of TNF‐a, LDH, MDA, and total protein (Suddek, 2014). A study described by El Gazzar and colleagues investigated ameliorative role of thymoquinone against allergic airway inflammation via hampering Th‐2 cytokine initiation, cell infiltration and hyperplasia, IL‐4, IL‐5, and IL‐13, and also initiated the IFN‐a production (El Gazzar et al., 2006). It also attenuated the inflammation via lowering the COX‐2 expression and PGD‐2 production (El Mezayen et al., 2006). In OVA experimental subjects, thymoquinone inhibited the lipoxygenase expressions, deteriorated the levels of LTB‐4 and LTC‐4, and lowered the Th2 cytokines (El Gazzar et al., 2006).

## 9 CONCLUSION

Thymoquinone is a phytochemical compound found in the plant *Nigella sativa*. Evidently, thymoquinone is chemically known as 2‐methyl‐5‐isopropyl‐1, 4‐benzoquinone, an active principal component of the volatile oil that exhibits wide spectrum of health‐endorsing properties such as anti‐inflammatory, hepatoprotective, antimicrobial, antitumor, antimutagenic, antiepileptic, neuroprotective, and nephroprotective, respectively. Health‐associated perspectives of this bioactive compound led to medical applications. In several anti‐inflammatory and degenerative disorders such as cancer, thymoquinone also has been known to modify the multiple molecular and signaling pathways. Most important aspects of thymoquinone such as hepatoprotective, anti‐inflammatory, and antiaging have been highlighted through various pathways, and further utilization of this compound in diet has been proven effective against different types of cancers.

## CONFLICT OF INTEREST

There is no conflict of interest among authors.

## ETHICAL APPROVAL

Not Applicable.

## Data Availability

Data available on request from the authors.
